# Evidence-based treatment strategies and a systematic review of AO/OTA type C2 intercondylar fractures of the distal humerus in pediatric and adolescent populations

**DOI:** 10.3389/fped.2026.1785713

**Published:** 2026-03-09

**Authors:** ZhaoHui Zhou, Xin Li, WeiHao Wang, Bo Jian, Fu Sai, MuHan Na, Fan Lu, YiShan Wei

**Affiliations:** 1 Inner Mongolia Medical University, Hohhot, Inner Mongolia Autonomous Region, China; 2 Children's Orthopedic Medical Center, The Second Affiliated Hospital of Inner Mongolia Medical University, Hohhot, Inner Mongolia Autonomous Region, China

**Keywords:** adolescents, children, humerus, intercondylar fractures, intra-articular elbow fractures, treatment

## Abstract

**Objective:**

This study seeks to assess the clinical outcomes associated with closed reduction with percutaneous pinning (CRPP) in comparison to open reduction and internal fixation (ORIF) for AO/OTA Type C2 distal humeral intercondylar fractures in pediatric and adolescent populations. A systematic review of the literature was performed to identify strategies that facilitate rapid recovery of elbow function and prevent complications.

**Methods:**

A retrospective analysis was conducted on 36 cases that were treated and followed up from November 2015 to September 2023. In addition, a systematic review of the literature was performed, covering the period from 1958 to 2025. Providing aggregated data on 62 pediatric patients. Radiological parameters, including the Baumann angle, lateral humeral condylar angle, and horizontal rotation rate, were measured using AutoCAD software. Postoperative functional outcomes were evaluated using the Visual Analog Scale (VAS), elbow range of motion, and the Mayo Elbow Performance Score (MEPS). Complications such as heterotopic ossification and trochlear necrosis were also monitored.

**Results:**

The mean follow-up duration was 24.71 ± 20.19 months, with all cases achieving clinical union within 6–8 weeks post-surgery. The CRPP group exhibited a significantly shorter operative time (34.28 ± 8.74 min vs. 54.67 ± 10.96 min) and reduced fluoroscopic exposure (24 ± 9 vs. 11 ± 3 instances) compared to the ORIF group (both *P* < 0.05). Radiological parameters, including the Baumann angle, lateral humeral condylar angle, and horizontal rotation rate, did not show statistically significant differences at 16 weeks post-surgery or at the final follow-up (*P* > 0.05). At the final follow-up, the CRPP group achieved significantly greater elbow flexion (139.72 ± 2.45° vs. 136.92 ± 3.06°) compared to the ORIF group (*P* < 0.05), while extension outcomes were comparable (*P* > 0.05). No significant differences were observed in VAS scores, MEPS scores, or complication rates between the two groups (*P* > 0.05).

**Conclusion:**

CRPP demonstrates non-inferiority to ORIF in achieving functional recovery, with advantages in operative efficiency for select cases.

**Clinical Trial Registration:**

http://www.medresman.org.cn/uc/projectsh/projectadd.aspx, Identifier ChiCTR2500113475.

## 1 Introduction

Distal humeral intercondylar fractures in pediatric and adolescent populations constitute a rare but significant injury, presenting complex therapeutic challenges and a substantial risk of long-term complications (1). These fractures are typically the result of high-energy trauma and can lead to a variety of complications, such as elbow joint stiffness, delayed union, or non-union of the fracture, all of which can considerably affect the long-term quality of life in affected children (2–4). It is crucial to differentiate between intercondylar fractures and extension-type supracondylar fractures, as the former involves a fracture line that extends into the intercondylar region of the distal humerus. Accurate diagnosis and effective treatment depend on high-quality radiographic imaging (e.g., x-rays or CT scans) and a thorough assessment of the trauma mechanism (5).

In the AO/OTA Type C classification system, distal humeral intercondylar fractures are categorized into three distinct types: Type C1 (Intercondylar Separation without Comminution), Type C2 (Metaphyseal Comminution with Intercondylar Displacement), Type C3 (Articular Surface Comminution with Intercondylar Separation). This classification is widely utilized and is particularly relevant for pediatric and skeletally immature patients with long bone fractures (6). Despite its clinical significance, the management of AO/OTA Type C2 intercondylar fractures in children and adolescents remains underreported, with a paucity of evidence-based strategies available for this uncommon fracture pattern. The selection of treatment is contingent upon the fracture pattern, patient age, and the surgeon's experience. Historically, open reduction and internal fixation (ORIF) has been the primary method employed to restore displaced intra-articular fragments (7, 8). However, open reduction can exacerbate the compromise of surrounding soft tissues, thereby elevating the risk of postoperative stiffness, a significant concern in skeletally immature populations. In contrast, distal humeral intercondylar fractures in pediatric and adolescent patients typically demonstrate reduced displacement and fragmentation, attributable to the thicker periosteum and abundant cartilage. These anatomical characteristics create favorable conditions for closed reduction with percutaneous pinning (CRPP) (9). Nevertheless, achieving accurate closed reduction of intra-articular fragments and obtaining clear fluoroscopic images continue to present technical challenges in clinical practice (10).

This retrospective study evaluates the surgical outcomes associated with closed and open reduction techniques for managing AO/OTA Type C2 intercondylar fractures of the distal humerus in pediatric and adolescent populations. The study has two primary objectives: first, to analyze, alongside a systematic literature review, the differences between these treatment modalities concerning intraoperative reduction quality, postoperative elbow function recovery, and complication rates specific to this fracture type; and second, to investigate potential future treatment directions for AO/OTA Type C2 distal humeral intercondylar fractures in children and adolescents.

## 2 Materials and methods

### 2.1 Inclusion and exclusion criteria

#### 2.1.1 Inclusion criteria

Age: Participants must be under 18 years.
Diagnosis: Participants must have confirmed AO/OTA Type C2 distal humeral intercondylar fractures, as verified through radiological imaging.
Surgical History: Participants must not have undergone any previous surgical intervention for distal humeral fractures.
Medical Records: Participants must have comprehensive preoperative and postoperative medical records, including radiological evaluations.
Follow-Up: Participants must have a follow-up period exceeding one year.

#### 2.1.2 Exclusion criteria

Pathological Fractures: Participants with distal humeral fractures resulting from primary or metastatic bone tumors, parathyroid disorders, or congenital bone diseases are excluded.
Severe Preexisting Joint Conditions: Participants with severe arthritic conditions that significantly impair elbow joint function and hinder the performance of activities of daily living (ADLs) are excluded.
Neuromuscular Limitations: Participants with conditions such as hemiplegia, myasthenia gravis, or other neuromuscular disorders that impede postoperative functional rehabilitation of the affected limb are excluded.
Preoperative Complications: Participants diagnosed with acute compartment syndrome of the forearm prior to surgery are excluded.

### 2.2 Baseline demographics and study population

**Figure 1:**
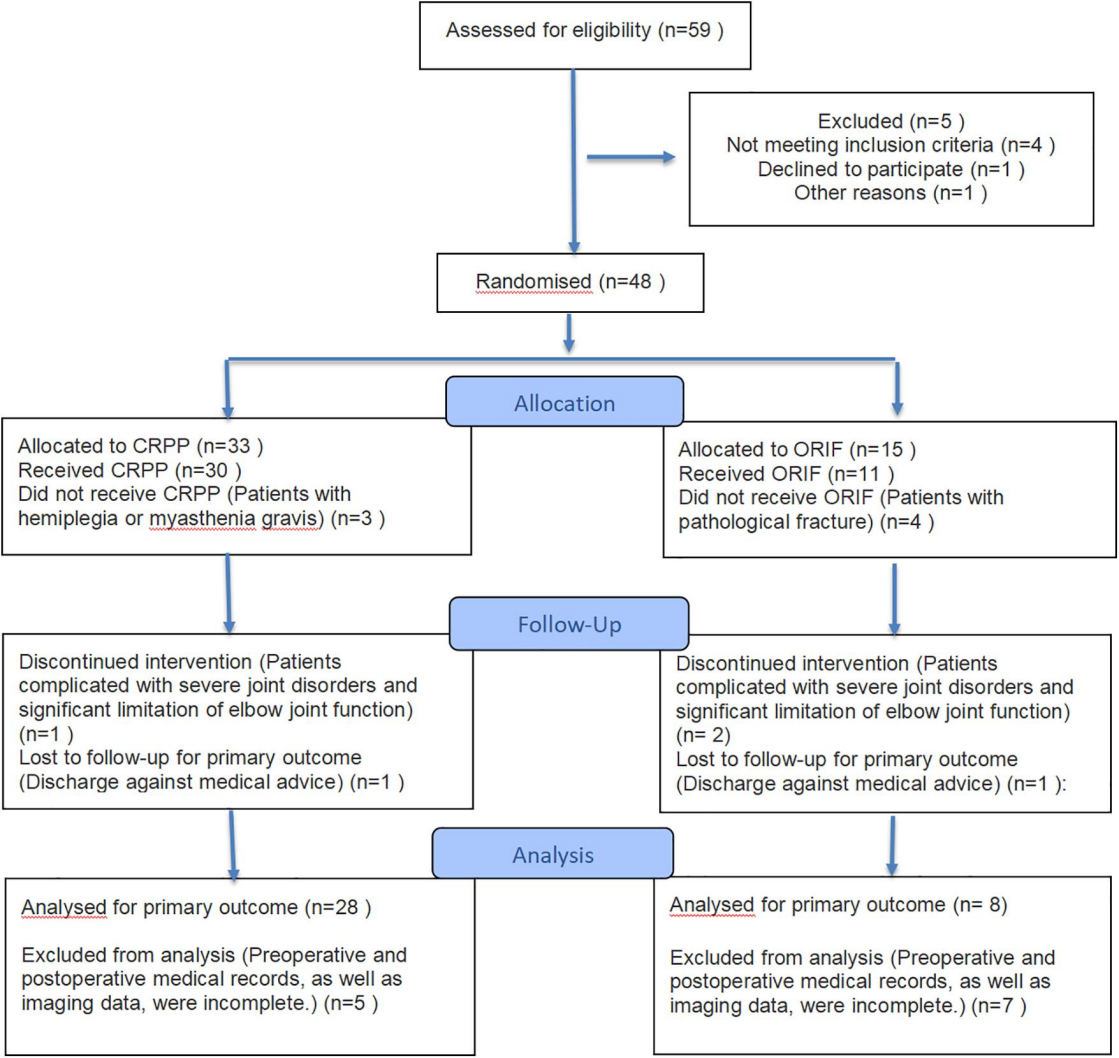
The progression of patients throughout the study (41).

In accordance with the predefined inclusion and exclusion criteria, a retrospective cohort study was conducted involving 36 pediatric and adolescent patients diagnosed with AO/OTA Type C2 distal humeral intercondylar fractures. These patients were treated at our center between November 2015 and September 2023. The cohort consisted of 24 male and 12 female participants, with a mean age at diagnosis of 11.78 ± 2.38 years. This study received approval from the Medical Ethics Committee of the Second Affiliated Hospital of Inner Mongolia Medical University (Approval Number: EFY20250136). Informed consent was obtained from the parents of all pediatric participants prior to the collection and analysis of data (Figure 1).

### 2.3 Treatment allocation criteria included

1) Fracture comminution degree/classification [assessed by computed tomography (CT)];
2) Soft tissue injury status (swelling, open injury);
3) Patient age and epiphyseal closure status;
4) Feasibility of reduction under intraoperative fluoroscopy.

Yang (11) reported that for AO/OTA type C1 and C2 fractures, the relatively simple fracture pattern allows for the attempt of closed reduction and internal fixation with lower risk. In contrast, AO/OTA type C3 fractures, characterized by severe comminution and complexity, typically require more direct open reduction and internal fixation to ensure therapeutic efficacy. Due to the retrospective design of this study, treatment allocation was determined individually by the surgical team based on the aforementioned clinical factors, which may introduce selection bias.

### 2.4 Data collection methods

The data collection methods employed in this study involved preoperative diagnostic imaging for all included patients, which comprised standard anteroposterior and lateral radiographs of the elbow. These were supplemented with computed tomography (CT) scans integrated with three-dimensional (3D) reconstruction to confirm AO/OTA Type C2 distal humeral intercondylar fractures. Magnetic resonance imaging (MRI) was selectively utilized to evaluate ligamentous injuries around the elbow joint when clinically indicated. Elbow radiological parameters and range of motion (ROM) measurements were quantified using AutoCAD software (Autodesk, USA), thereby ensuring standardized and reproducible data acquisition.

### 2.5 Study design and methodology

The study employed a comprehensive methodology that combined clinical data analysis with a systematic review of the literature. This approach included the following components: 1. Statistical Variables: Demographic and clinical data were collected and analyzed, encompassing variables such as gender, age, side of injury, treatment modality, duration of follow-up, postoperative elbow function, and complication rates. 2. Group Comparisons: Patients were stratified based on their treatment approaches, and clinical outcomes were statistically compared across these groups. 3. Literature Screening: Studies retrieved from the literature search underwent an initial screening process based on their titles and abstracts. This was followed by a full-text review, adhering to predefined inclusion and exclusion criteria. Data from the selected studies, along with patient records, were systematically organized in Excel spreadsheets for subsequent statistical analysis. 4. Data Handling: To ensure data integrity, variables that were marked with a “?” in the literature, indicating insufficient or absent descriptions, were excluded from the statistical analysis.

#### 2.5.1 Surgical techniques: closed reduction and percutaneous pinning (CRPP)

All patients underwent CRPP under either general anesthesia or brachial plexus block. The procedure adhered to a standardized four-step protocol: (1) traction and reduction, (2) alignment of the smaller fragment to the larger one, optionally employing a levering technique, (3) conversion from intercondylar to supracondylar alignment, and (4) supracondylar fixation.

**Figure 2:**
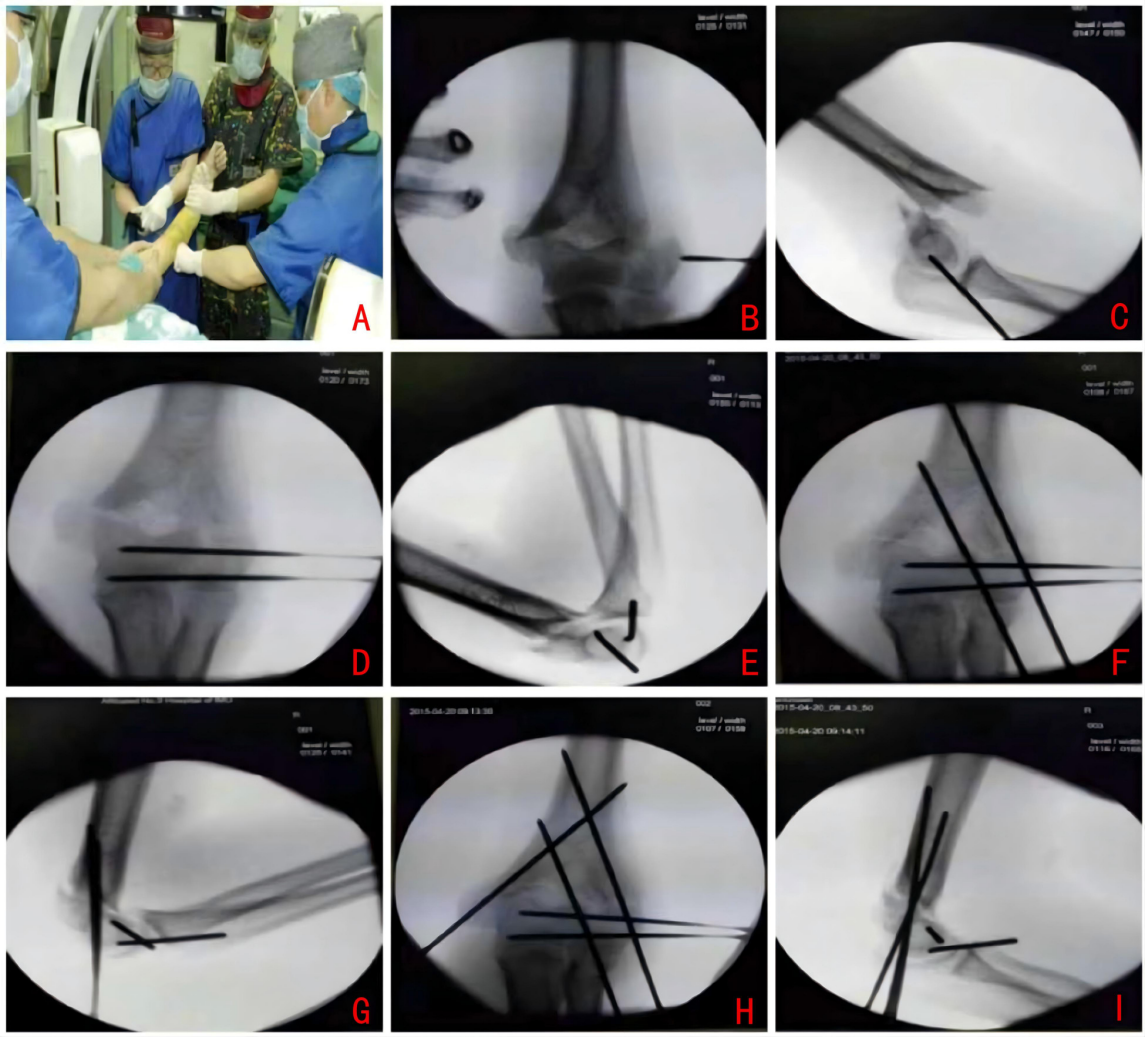
**(A)** Traction reduction. **(B,C)** Reduction via the “Leverage Principle” (Joystick Technique). **(D,E)** Conversion from intercondylar to supracondylar fracture pattern. **(F,G)** Supracondylar fixation. **(H,I)** Immediate postoperative radiographs.

Step 1: Traction and Reduction Following the induction of adequate anesthesia, patients were positioned supine with the affected shoulder abducted to 90 degrees. Longitudinal traction was applied to the elbow, which was partially flexed. A C-arm fluoroscope or x-ray machine was aligned parallel to the operating table, with the image receptor situated directly beneath the abducted elbow (refer to Figure 2A).
Step 2: Alignment of the Smaller Fragment to the Larger Fragment (Optional Levering Technique) The smaller intercondylar fragment was manually aligned with the larger fragment to achieve initial alignment (Figure 2B). Under fluoroscopic guidance, with adjustments to the C-arm while maintaining the limb's position, reductions in the coronal and sagittal planes at the intercondylar region were verified. A 2.0 mm Kirschner wire (K-wire) was utilized as a “joystick” to aid in the levering and compression of the intercondylar fracture (Figure 2C).
Step 3: Conversion from Intercondylar to Supracondylar Configuration Upon achieving satisfactory intercondylar reduction, 1–2 K-wires, each 2.0 mm in diameter, were inserted parallel to the plane of the elbow joint to stabilize the reduced intercondylar fragment (Figures 2D,E). This procedure effectively transformed the intercondylar fracture into a supracondylar configuration to facilitate subsequent fixation.
Step 4: Supracondylar Fixation The supracondylar fracture was closed-reduced, with alignment in both the coronal and sagittal planes verified fluoroscopically. A single 2.0 mm Kirschner wire (K-wire) was inserted from the lateral epicondyle to engage the medial cortex, with fluoroscopic imaging in the coronal plane confirming the initial pin trajectory (Figure 2F). A second K-wire was inserted parallel to the first through the medial epicondyle (Figure 2H). Fluoroscopic confirmation of anatomical reduction in both the coronal and sagittal planes was conducted (Figures 2H,I).
Step 5: Intraoperative Management The K-wires were bent and trimmed, with the wire ends positioned externally. The elbow was immobilized at 90° of flexion in a supracondylar cast with foam padding to minimize soft tissue compression. The forearm was maintained in a neutral or slightly supinated position (12).

#### 2.5.2 Open reduction and internal fixation (ORIF)

In the treatment of AO/OTA Type C2 distal humeral intercondylar fractures in pediatric and adolescent patients, Open Reduction and Internal Fixation (ORIF) was conducted using three standardized surgical approaches: medial, lateral, and posterior (36). The posterior approach, which frequently involves olecranon osteotomy, is described in detail as follows (37):

Incision and Exposure: Following the administration of adequate anesthesia, a longitudinal incision measuring 6–8 cm was made along the posterior midline of the elbow. Layered dissection was performed to expose and protect the ulnar nerve sequentially.
Olecranon Osteotomy and Intercondylar Exposure: The surrounding muscle and tissues were dissected in layers. A V-shaped osteotomy was created 2–3 cm proximal to the olecranon tip within the avascular zone. Subsequently, the osteotomized fragment was flipped to allow for exposure of the intercondylar fracture.
Fracture Reduction and Fixation: Reduction forceps were employed to grasp the medial and lateral epicondyles, thereby correcting the separation and displacement. Under direct visualization, the fracture was confirmed to be anatomically reduced and subsequently fixed using cortical screws and medial/lateral plates for both intercondylar and supracondylar fractures. The olecranon osteotomy site was similarly reduced and stabilized using Kirschner wires (K-wires) in conjunction with tension band wiring (13).
Hemostasis and Closure: Adequate hemostasis was achieved, followed by a layered closure of the incision. The elbow was externally immobilized with a cast.

### 2.6 Postoperative management

Within three days following surgery, all patients received anteroposterior and lateral radiographic evaluations of the elbow. The initial outpatient follow-up was scheduled for three weeks post-surgery, during which splints were removed and one radial K-wire was extracted, contingent upon radiographic evidence of callus formation at this juncture. Guided gentle elbow mobilization exercises were then commenced. The complete removal of all K-wires was executed in stages between six to eight weeks postoperatively, depending on the presence of radiographic bridging callus (14).

### 2.7 Evaluation criteria

#### 2.7.1 Radiographic evaluation

All radiographic parameters were measured by an independent orthopedic surgeon using AutoCAD

**Figure 3:**
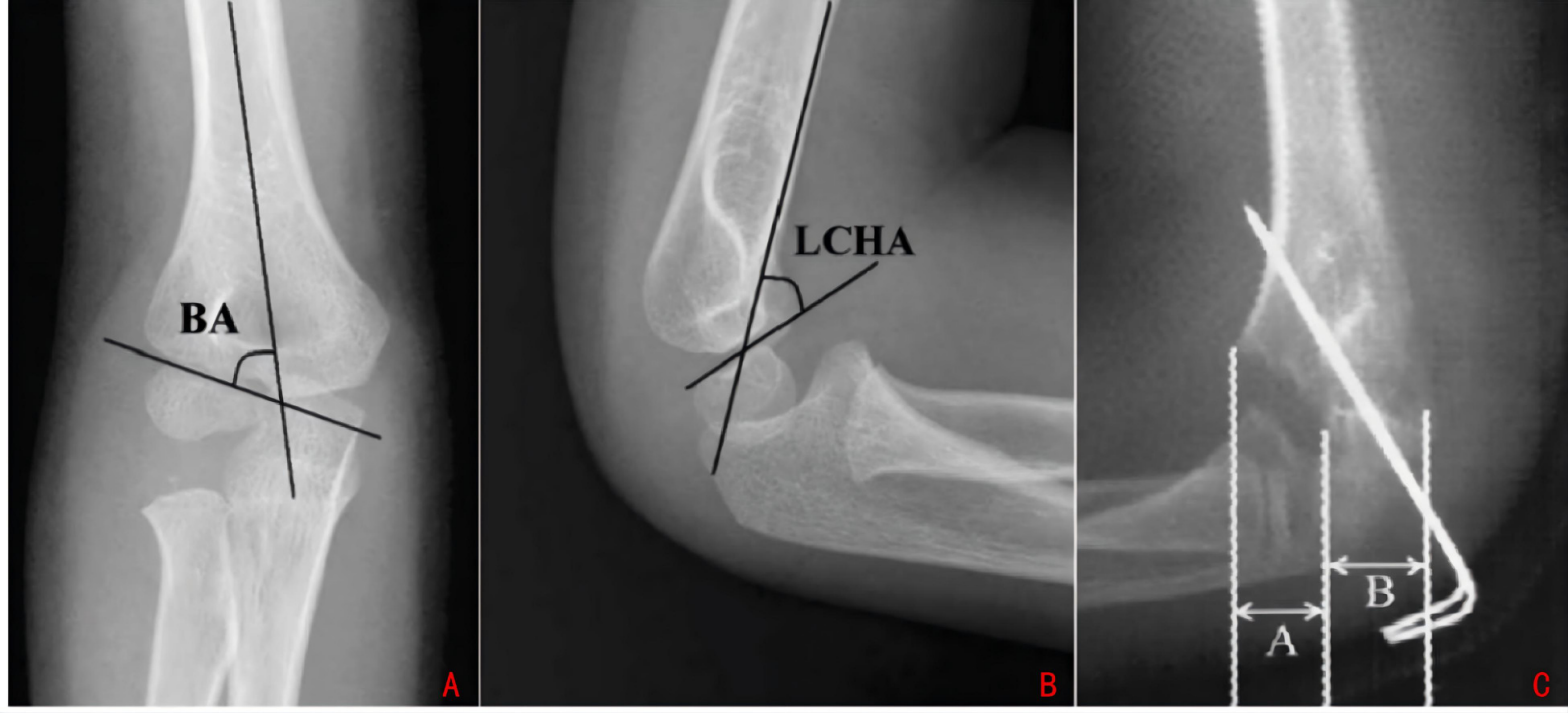
**(A)** Baumann's angle. **(B)** Lateral humerocapitellar angle. **(C)** Humeral rotational alignment ratio.

Baumann's Angle: Baumann's angle is defined as the angle formed between the lateral epiphyseal line of the distal humerus and the longitudinal axis of the humerus, as observed on a standard coronal radiograph of the elbow. The normal reference range for this angle is between 75° and 80° (refer to Figure 3A).
Lateral Humeral Epiphyseal Angle (LHEA): The lateral humeral epiphyseal angle is determined by the intersection of the anterior cortex line of the distal humerus and the distal humeral epiphyseal line, as seen on a standard sagittal radiograph of the elbow. The normal mean value for this angle is 51° (refer to Figure 3B).
Horizontal Rotational Rate: The horizontal rotational rate is calculated by measuring the displacement (A) on the lateral radiograph of the elbow, dividing it by the distal humeral width (B) at the fracture level, and then multiplying the result by 100 to express it as a percentage (refer to Figure 3C).

#### 2.7.2 Functional outcome measures

Elbow function was evaluated utilizing the Mayo Elbow Performance Score (MEPS) (15), while pain levels were assessed through the Visual Analog Scale (VAS) (16). The MEPS is a widely recognized instrument for assessing elbow joint outcomes, encompassing four domains: pain (45 points), range of motion (20 points), stability (10 points), and activities of daily living (25 points). The cumulative score ranges from 0 to 100, with classifications as excellent (≥90 points), good (75–89 points), fair (60–74 points), or poor (<60 points). Pain intensity was quantified using the VAS, with scores interpreted as follows: 0 (no pain), 1–3 (mild pain), 4–6 (moderate pain), and 7–10 (severe pain).

### 2.7 Statistical analysis

Statistical analyses were conducted using SPSS version 26.0 (IBM Corp., USA). Continuous variables are reported as mean ± standard deviation (x¯ ± s). The Shapiro–Wilk test was employed to assess normality; independent samples *t*-tests were utilized for data following a normal distribution, while Mann–Whitney *U*-tests were applied for non-normally distributed data. Categorical data are presented as percentages, and group comparisons were performed using chi-square tests.

## 3 Results

The study comprised 36 pediatric patients diagnosed with AO/OTA Type C2 distal humeral intercondylar fractures, including 24 males and 12 females. Fractures occurred on the left side in 22 cases (61.1%) and on the right side in 14 cases (38.9%). Twenty-eight patients underwent closed reduction and percutaneous pinning (CRPP), whereas eight patients received open reduction and internal fixation (ORIF). No significant differences were found between the groups concerning gender, age, fracture type, affected side, or follow-up duration (all *P* > 0.05), thereby confirming the comparability of the groups. The baseline demographic analysis of the patients is detailed in Table 1. Intraoperative data are detailed in Table 2. The comparison of elbow joint imaging data is detailed in Table 3. The CRPP group demonstrated significantly shorter operative times (34.28 ± 8.74 min) but required a greater number of fluoroscopic exposures (24 ± 9) compared to the ORIF group (54.67 ± 10.96 min and11 ± 3exposures, respectively). All fractures achieved clinical and radiographic healing by 8 weeks postoperatively.

**Table 1:** Summary of baseline demographic and clinical characteristics in pediatric and adolescent patients with AO/OTA type C2 distal humeral intercondylar fractures.

Variable	CRPP group (*n* = 28)	ORIF group (*n* = 8)	X2/*z*-value	*P*-value
Gender				
Male	17 (60.7%)	7 (87.5%)	2.009	0.156
Female	11 (39.3%)	1 (12.5%)		
Affected Side				
Left	17 (60.7%)	5 (62.5%)	0.008	0.927
Right	11 (39.3%)	3 (37.5%)		
Age at Diagnosis (years)	12.0 (11.0, 14.0)	11.5 (8.3, 14.0)	−0.583	0.56
Follow-up (months)	13.0 (12.0, 24.0)	12.0 (12.0, 22.5)	−0.551	0.582

**Table 2:** Comparison of intraoperative data between CRPP and open reduction groups for AO/OTA type C2 distal humeral intercondylar fractures in pediatric patients.

Group	Incision Length (cm)	Fluoroscopy Times (times)	Acute VAS Score	Recovery VAS Score	Operative Time (min)	Intraoperative Blood Loss (ml)	Fracture Healing Time (weeks)
CRPP group (*n* = 28)	1 (1, 1.2) [Median (IQR)]	24 ± 9 [Mean ± SD]	6 (5, 6) [Median (IQR)]	2 (1, 2) [Median (IQR)]	34.28 ± 8.74 [Mean ± SD]	16.25 ± 9.68 [Mean ± SD]	6.51 ± 1 [Mean ± SD]
Open reduction group (*n* = 8)	8 (4, 11.96) [Median (IQR)]	11 ± 3 [Mean ± SD]	6 (5, 6) [Median (IQR)]	2 (1, 2) [Median (IQR)]	54.67 ± 10.96 [Mean ± SD]	69.29 ± 17.42 [Mean ± SD]	8.79 ± 1.04 [Mean ± SD]
Statistical test	Z = −4.082	Z = −4.049	Z = −0.353	Z = −0.135	T = 0.953	T = 10.928	T = 5.347
*P*-value	<0.001*	<0.001*	0.724	0.893	0.347	<0.001*	<0.001*

*Z*-test (non-parametric) was used for ordinal data (VAS scores), and *T*-test (parametric) was used for continuous data.

* Denotes statistically significant differences (*P* < 0.05).

**Table 3:** Comparative analysis of imaging parameters at three time points for AO/OTA type C2 distal humeral intercondylar fractures in children and adolescents treated with open vs. Closed Reduction.

Parameter/Time Point	Open reduction group (*n* = 8)	Closed reduction group (*n* = 28)	Test statistic (t/z)	*P*-value
Coronal Plane				
• Intraoperative Baumann angle (°)	74.39 ± 3.24	73.97 ± 3.24	−0.165	0.869
• Baumann angle at 16 weeks postoperatively (°)	74.13 ± 1.95	73.77 ± 1.99	−0.454	0.65
• Baumann angle at final follow-up (°)	73.81 ± 1.30	74.15 ± 2.77	−0.481	0.635
Sagittal Plane				
• Intraoperative lateral humeral-capitellar angle (°)	52.66 ± 1.94	53.35 ± 2.37	−0.713	0.481
• Lateral humeral-capitellar angle at 16 weeks postoperatively (°)	52.14 ± 1.18	53.18 ± 1.84	−1.416	0.166
• Lateral humeral-capitellar angle at final follow-up (°)	51.32 ± 3.65	53.28 ± 2.18	−1.849	0.073
Horizontal Plane				
• Intraoperative reduction rate (%)	98.92 ± 7.14	105.87 ± 5.09	−1.979	0.05

At the final follow-up, all patients demonstrated good to excellent functional outcomes as assessed by the Mayo Elbow Performance Score (MEPS), with a mean score of 88.43 ± 7.80 points. Pain scores showed a significant reduction from the acute phase [Visual Analog Scale (VAS): 6 (5, 6)] to the recovery phase [VAS: 2 (1, 2)]. At the 16-week mark, the CRPP group exhibited elbow flexion and extension measurements of 137.23 ± 5.90° and −2.52 ± 6.79°, respectively, compared to 131.98 ± 5.02° and 0.06 ± 3.97° in the ORIF group. By the final follow-up, these measurements improved to 139.72 ± 2.45° flexion and 0.78 ± 4.15° extension in the CRPP group, and 136.92 ± 3.06° flexion and 2.56 ± 0.85° extension in the ORIF group. A statistically significant difference in flexion outcomes was observed between the groups (*P* < 0.05), whereas extension outcomes were not significantly different (*P* > 0.05). The range of motion of the elbow joint after the operation is detailed in Table 4. Sun (17) demonstrated that the minimal clinically important difference (MCID) for elbow flexion is typically 25°. Although a statistically significant difference was observed, a 3° change in elbow flexion may not reach this MCID threshold, and thus the findings require cautious interpretation. Four complications were documented: three in the CRPP group, including joint stiffness, pin breakage, and trochlear necrosis, and one instance of heterotopic ossification in the ORIF group. All complications were effectively managed, resulting in no long-term functional impairments. Notably, trochlear necrosis resolved almost completely by 13 months, with no radiographic signs of growth arrest or avascular necrosis.

**Table 4:** Comparison of postoperative elbow range of motion in children and adolescents with AO/OTA type C2 distal humeral intercondylar fractures.

Measurement	Open Reduction (*n* = 8)	Closed Reduction (*n* = 28)	*t*-value	*P*-value
Flexion, Affected Side, 16-week FU	131.98 ± 5.02°	137.23 ± 5.90°	−2.16	0.038*
Flexion, Contralateral Side, 16-week FU	143.44 ± 4.46°	141.75 ± 3.46°	1.093	0.282
Extension, Affected Side, 16-week FU	0.06 ± 3.97°	−2.52 ± 6.79°	0.955	0.346
Extension, Contralateral Side, 16-week FU	2.89 ± 1.43°	2.36 ± 1.30°	0.957	0.345
Flexion, Affected Side, Final FU	136.92 ± 3.06°	139.72 ± 2.45°	−2.573	0.015*
Flexion, Contralateral Side, Final FU	143.73 ± 4.70°	141.96 ± 3.64°	1.081	0.287
Extension, Affected Side, Final FU	2.56 ± 0.85°	0.78 ± 4.15°	1.114	0.273
Extension, Contralateral Side, Final FU	3.04 ± 1.19°	2.61 ± 1.47°	0.714	0.48

*Denotes statistically significant differences (*P* < 0.05).

## 4 Discussion

### 4.1 Literature search strategy

A comprehensive literature search was systematically conducted utilizing the following keywords: “intercondylar fractures of the humerus in children”, “intercondylar fractures of the humerus in adolescents”, “distal intercondylar fracture of the humerus in children”, “distal intercondylar fracture of the humerus in adolescents”, “distal humeral T-shaped intercondylar fracture in children”, and “distal humeral T-shaped intercondylar fracture in adolescents”. The databases searched included CNKI (China National Knowledge Infrastructure), Wanfang Data, VIP Database, PubMed, and Web of Science, covering the period from 1958 to 2025. Inclusion Criteria: 1. Full-text articles explicitly addressing intercondylar fractures of the distal humerus in pediatric or adolescent populations. 2. Retrospective series or clinical studies concentrating on distal humeral intercondylar fractures in children and adolescents.

**Figure 4:**
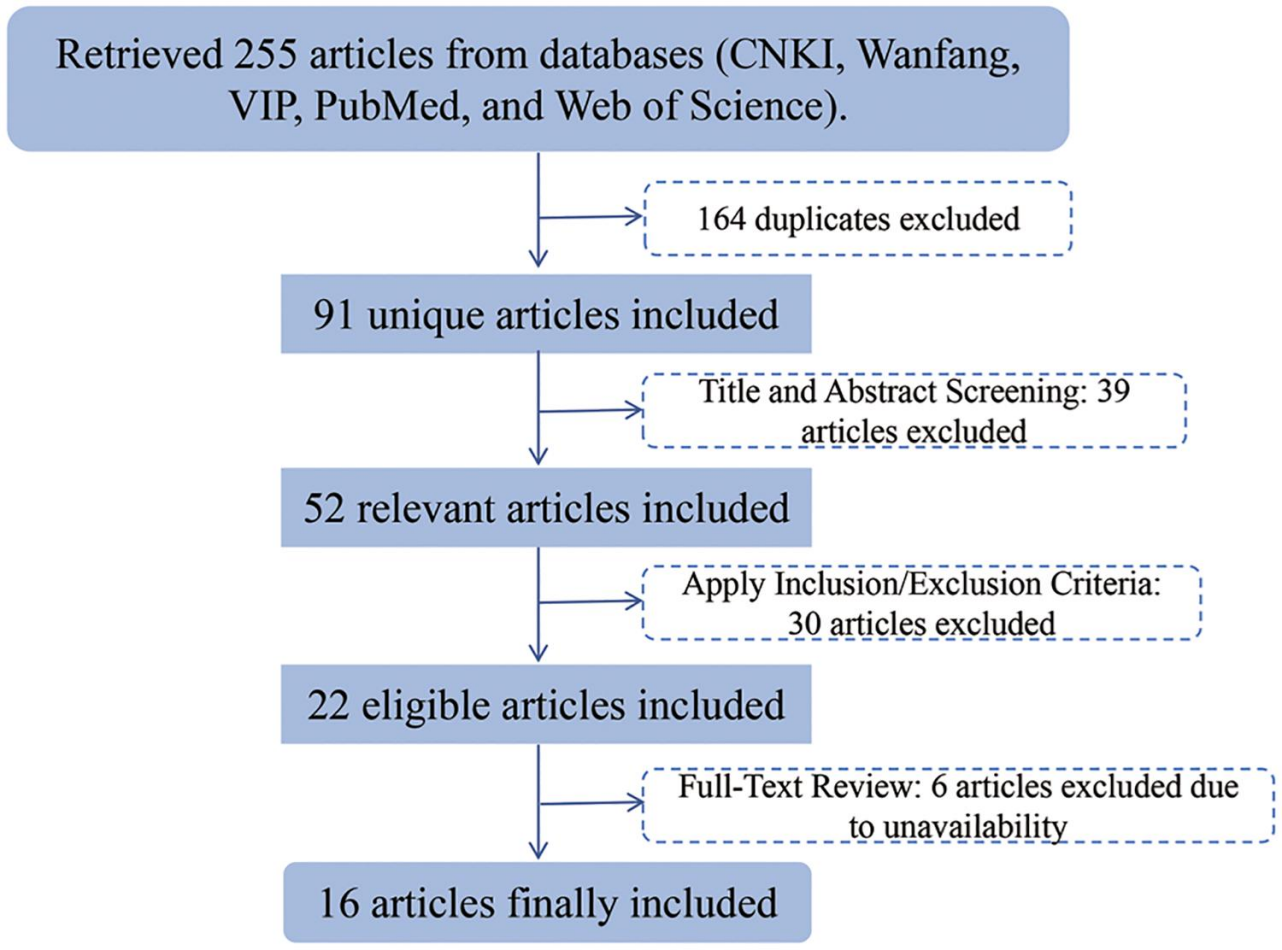
Flow diagram of the literature screening process.

Exclusion Criteria: 1. Articles for which the full text concerning distal humeral intercondylar fractures in children and adolescents could not be obtained. 2. Studies that do not include radiographic data on distal humeral intercondylar fractures in pediatric and adolescent patients. 3. Duplicate or redundant publications on the same type of fracture. An initial screening of titles and abstracts identified 52 relevant articles. Following a comprehensive full-text review and the application of inclusion and exclusion criteria, six articles were excluded due to the unavailability of full text. The final analysis comprised 16 peer-reviewed articles, including 13 in English and 3 in Chinese, which encompassed case reports, clinical trials, and retrospective series on distal humeral intercondylar fractures in pediatric and adolescent populations (Figure 4).

### 4.2 Search results

#### 4.2.1 General findings

A comprehensive systematic review was conducted on all published literature concerning distal intercondylar fractures of the humerus in pediatric and adolescent populations, spanning the years 1958–2025. This review was supplemented with the authors' own case data to provide a summary of baseline patient characteristics. Among the 16 articles included in the review, which encompassed a total of 62 cases, all patients underwent surgical intervention. Specifically, 11 patients (17.7%) received closed reduction with percutaneous pinning (CRPP), while 51 patients (82.2%) were treated with open reduction and internal fixation (ORIF). No statistically significant differences were identified between the CRPP and ORIF groups regarding gender, age, affected side, fracture classification, radiographic findings, or follow-up duration. This lack of significant differences suggests that the baseline characteristics were homogeneous, thereby ensuring comparability between the groups (Table 5).

**Table 5:** Literature summary of clinical cases with AO/OTA type C2 distal humeral intercondylar fractures in pediatric patients.

Case	Author (year)	Gender	Age at Diagnosis (years)	Affected Side	Treatment Method (O: Open Reduction, C: CRPP)	Mean Follow-up Time (months)	Functional Outcome (Excellent/Good/Fair)
1	Author	Male (24), Female (12)	11.77 ± 2.37	Left (22), Right (14)	O: 8, C: 28	22.31	Excellent: 30, Good: 6, Fair: 0
2	Jarvis JG (1984) (18)	Male and Female (12)	12.75 ± 1.99	Not reported (12)	O: 12, C: 0	32	Excellent: 6, Good: 4, Fair: 2
3	VasiliosA (1986) (8)	Male (4), Female (1)	10.40 ± 1.85	Not reported (5)	O: 5, C: 0	60	Excellent: 4, Good: 1, Fair: 0
4	RuizAL (2001) (5)	Male	6	Left	O: 0, C: 1	24	Excellent
5	KanellopoulosAD (2004) (19)	Male (2)	12.00 ± 1.00	Right (2)	O: 0, C: 2	14	Excellent: 2, Good: 0, Fair: 0
6	OsadaD (2005) (20)	Female	3	Left	O: 1, C: 0	36	Good
7	Wu Yongtao (2011) (3)	Male (5), Female (1)	8.50 ± 1.91	Not reported (6)	O: 6, C: 0	26.4	Excellent: 5, Good: 1
8	Tang Yingming (2012) (21)	Male (5)	15.00 ± 1.41	Not reported (5)	O: 5, C: 0	20	Excellent: 4, Good: 0, Fair: 1
9	Mok CY (2013) (22)	Male (1), Female (2)	9.33 ± 1.15	Right (1), Left (2)	O: 3, C: 0	Not reported	Excellent: 1, Good: 2, Fair: 0
10	PantA (2013) (14)	Male (6), Female (1)	14.00 ± 0.82	Right (1), Left (6)	O: 7, C: 0	24	Excellent: 1, Good: 6, Fair: 0
11	Ducic S (2014) (13)	Male and Female (4)	13.88 ± 0.85	Not reported (4)	O: 0, C: 4	59.25	Excellent: 3, Good: 1, Fair: 0
12	TomoriY (2017) (23)	Male	7	Left	O: 1, C: 0	13	Excellent
13	Marengo L (2018) (24)	Male (7), Female (2)	11.61 ± 2.19	Right (4), Left (5)	O: 9, C: 0	40.8	Excellent: 4, Good: 5, Fair: 0
14	Salvador J (2020) (25)	Female (2)	7.50 ± 0.50	Right (1), Left (1)	O: 2, C: 0	24	Excellent: 2, Good: 0, Fair: 0
15	Gong Wei (2021) (28)	Female	12	Left	O: 0, C: 1	20	Excellent
16	Wen Shu (2022) (26)	Male	9	Not reported	O: 0, C: 1	39	Excellent
17	Yu Lu (2023) (9)	Female (2)	13.00 ± 1.00	Right (1), Left (1)	O: 0, C: 2	28	Excellent: 2, Good: 0, Fair: 0

#### 4.2.2 Treatment strategies and complications

##### 4.2.2.1 Treatment grouping

Based on the treatment strategies delineated in existing literature, supplemented by the authors' clinical experience, 98 pediatric and adolescent patients with distal intercondylar humeral fractures were categorized into two cohorts:

the CRPP Group, consisting of 39 patients (23 males and 16 females) with a mean age at diagnosis of 12.01 ± 1.98 years and a mean follow-up duration of 27.45 ± 21.84 months; and the ORIF Group, comprising 59 patients (43 males and 16 females) with a mean age at diagnosis of 11.41 ± 3.05 years and a mean follow-up duration of 31.26 ± 25.78 months.

#### 4.2.3 Radiographic outcomes before and after treatment

**Figure 5:**
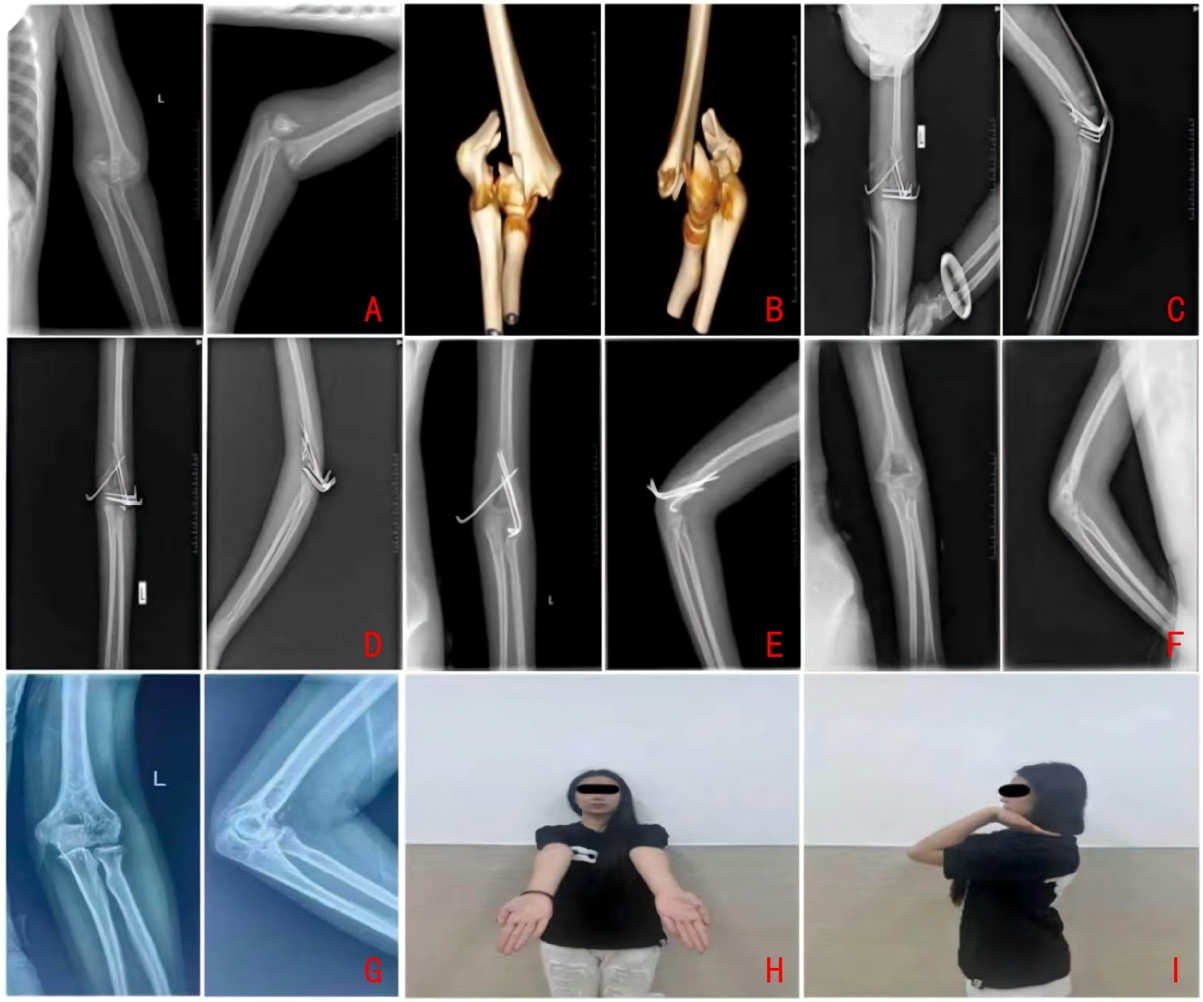
Illustrative case radiographic series of a pediatric distal humeral intercondylar fracture treated in this study. **(A)** Preoperative anteroposterior and lateral radiographs. **(B)** Preoperative CT with 3D reconstruction. **(C)** Intraoperative anteroposterior and lateral views under fluoroscopic guidance. **(D)** Postoperative radiographs at 4 weeks. **(E)** Postoperative radiographs at 8 weeks. **(F)** Postoperative radiographs at 16 Weeks. **(G)** Radiographs at final follow-up. **(H)** Clinical appearance at final follow-up.

Elbow extension limitation was defined as the angle between 0° and the maximum extension achieved on the injured side, whereas flexion limitation was quantified as the difference between the maximum flexion of the injured side and that of the contralateral, uninjured side. Elbow stiffness was classified as a flexion angle of less than 120° or an extension limitation exceeding 30° (39). Importantly, the study found that the ORIF group exhibited a poorer recovery of elbow flexion function in comparison to the CRPP group (Figure 5).

### 4.3 Complication profile

Intercondylar fractures of the humerus in pediatric and adolescent populations are uncommon injuries, generally associated with poorer clinical and functional outcomes compared to other pediatric humeral or elbow fractures (Table 6). These fractures are associated with a higher complication rate, particularly in patients over 10 years of age, where the risk of functional impairment—characterized by loss of elbow mobility—becomes more pronounced (24).

**Table 6:** Comparison of postoperative complication rates in children and adolescents with AO/OTA type C2 distal humeral intercondylar fractures.

Complication type	Open reduction (*n* = 59)	CRPP (*n* = 39)
Total cases	59/98 (60.20%)	39/98 (39.80%)
Joint stiffness	4/59 (6.78%)	1/39 (2.56%)
Cubitus varus deformity	4/59 (6.78%)	0
Implant failure (pin breakage)	0	1/39 (2.56%)
Trochlear necrosis	2/59 (3.39%)	1/39 (2.56%)
Heterotopic ossification	2/59 (3.39%)	0
Pin tract infection	1/59 (1.69%)	0
Transient median nerve palsy	1/59 (1.69%)	0
Cubitus valgus deformity	1/59 (1.69%)	0
Humeroradial joint osteoarthritis	1/59 (1.69%)	0

Through an extensive literature review, this study delineates three principal challenges in diagnosing and treating pediatric and adolescent humeral intercondylar fractures: (a) attaining an accurate diagnosis; (b) the absence of a universally applicable classification system, given that the widely utilized Gartland classification does not address this specific fracture type, with the AO/OTA classification potentially offering greater suitability for pediatric and adolescent cases (24, 27, 28); and (c) formulating effective treatment strategies that ensure precise reduction and stable fixation of fracture fragments to optimize clinical outcomes.

#### 4.3.1 Epidemiological characteristics of distal humeral intercondylar fractures in pediatric and adolescent populations

Rarity: Distal intercondylar fractures of the humerus in children and adolescents are relatively rare, comprising less than 1% of all pediatric elbow fractures Beghin et al. (27, 38). The presence of extensive cartilaginous structures and incomplete ossification in the distal humerus of skeletally immature patients complicates diagnosis and may lead to frequent underdiagnosis. An initial history of high-energy trauma can serve as a valuable indicator for identifying these injuries (40).
Mechanism of Injury: Evans (29) proposed that the etiology of these fractures involves an upward-directed force acting on the ulnar olecranon, which functions as a wedge to split and displace the humeral intercondylar region. This biomechanical mechanism results in the distal humerus being characterized by distinctive horizontal and vertical fracture lines. Maylahn (30), in his 1958 report on pediatric intercondylar fractures, emphasized that the extension of the fracture line into the intercondylar region is a critical diagnostic indicator for this type of injury.
Two classification systems are frequently utilized for distal humeral intercondylar fractures. The Toniolo Classification, as referenced in source (31), categorizes T-shaped intercondylar fractures into three subtypes based on the severity of displacement: Type I includes fractures with minimal displacement; Type II encompasses displaced but non-comminuted fractures; and Type III involves displaced and comminuted fractures. Nonetheless, when compared to the modified Arbeitsgemeinschaft für Osteosynthesefragen/Orthopaedic Trauma Association (AO/OTA) classification, the Toniolo system lacks the necessary granularity to effectively differentiate subtle variations in pediatric and adolescent intercondylar fractures. The Modified AO/OTA Classification system is widely recognized for its enhanced clinical applicability in pediatric and adolescent cases, owing to its anatomical precision and adaptability to immature bone structures. It categorizes all distal humeral intercondylar fractures into three distinct types based on the extent of articular involvement: Type C1 (Intercondylar Separation without Comminution),Type C2 (Metaphyseal Comminution with Intercondylar Displacement),Type C3 (Articular Surface Comminution with Intercondylar Separation). This classification has been revised to more effectively address the unique biomechanical and developmental challenges presented by skeletally immature patients (6).

#### 4.3.2 Historical evolution of treatment methods

Over the past fifty years, the management of distal humeral intercondylar fractures in pediatric and adolescent populations has followed a complex and often contentious path (32). A longstanding debate persists regarding the choice between conservative therapy and aggressive open reduction. Furthermore, identical treatment approaches can result in significantly different outcomes across various hospitals and practitioners, highlighting the critical importance of accurately identifying the indications for each strategy and executing interventions with technical precision. Successful outcomes for these fractures are predicated on three fundamental principles: the anatomic reduction of the articular surface, stable internal fixation of the fracture fragments, and early postoperative functional rehabilitation. These principles are not only essential for optimizing functional recovery in distal humeral intercondylar fractures but are also universally acknowledged as the gold standard for managing intra-articular fractures.

In the early literature, open reduction and internal fixation (ORIF) was the predominant treatment strategy for pediatric and adolescent distal humeral intercondylar fractures (27). Studies from this era (13, 18, 20) highlighted ORIF as a method to restore the integrity of the articular surface and ensure stable fixation of fracture fragments. However, this approach was associated with considerable risks of complications, including nonunion, delayed union, avascular necrosis, epiphyseal growth arrest, median nerve injury, heterotopic ossification, elbow stiffness, and post-traumatic osteoarthritis (28). Morrey (33) characterized elbow stiffness based on a loss of range of motion (ROM), defining it as an extension loss greater than 30° or a flexion loss less than 130°. Elbow stiffness was identified as a significant complication in the management of intercondylar fractures (34). In a case series involving 18 patients, Marengo L (24) reported that 14 cases were treated with open reduction and internal fixation (ORIF), with significant complications observed, including two instances of median nerve injuries, two cases of cubitus varus (elbow varus deformity), one case of cubitus valgus (elbow valgus deformity), and four cases exhibiting a loss of elbow motion of 20° or more. Notably, all these cases involved children over the age of 10, highlighting the increased risk of complications in older pediatric and adolescent populations undergoing surgical intervention. Re (7) conducted a retrospective analysis of 12 pediatric and adolescent cases managed with ORIF. However, all 12 patients demonstrated a partial loss of elbow range of motion (ROM) at the final follow-up, with an average extension loss of 13° (range: 5°–30°). These findings are consistent with Jarvis' functional classification criteria, which categorize outcomes as excellent (ROM loss <15°), good (ROM loss between 15° and 30°), and poor (ROM loss >30°) (18, 35).

Following 2015, advancements in the understanding of the mechanics of distal humeral intercondylar fractures, along with improvements in intraoperative closed reduction techniques and imaging modalities, have facilitated the adoption of percutaneous fixation as a viable clinical option (8, 24, 26). This minimally invasive approach reduces tissue trauma and accelerates elbow recovery, offering distinct advantages over traditional open reduction methods. Research by Pant A (14) underscores that closed reduction combined with percutaneous Kirschner wire (K-wire) fixation minimizes iatrogenic soft tissue injury, thereby potentially reducing the risk of post-traumatic elbow stiffness. Unlike large-scale internal fixation devices, this technique avoids complications such as hardware prominence, soft tissue irritation, or skin necrosis. Additionally, the percutaneous K-wires can be easily removed in an outpatient setting, obviating the need for secondary surgical interventions.

This approach substantially diminishes surgical and anesthetic risks for pediatric patients while concurrently reducing healthcare expenditures. Moreover, the duration of the procedure for closed reduction is considerably shorter than that of open reduction, thereby enhancing operational efficiency. Ducic S (13) advocates for a minimally invasive technique in skeletally immature patients, highlighting the use of percutaneous K-wire fixation to achieve stable fracture immobilization. This method prioritizes the prevention of additional tissue damage—and its associated risks, such as stiffness—while adhering to fundamental principles of intra-articular fracture management: achieving anatomical reduction of the distal humeral articular surface and ensuring secure fixation of fracture fragments.

#### 4.3.3 Emerging trends in treatment

A systematic literature review has identified an increasing trend in the use of closed reduction and percutaneous Kirschner wire internal fixation (CR-PKIF) since 2015. Specifically, data prior to 2015 indicated 39 cases treated with open reduction compared to only 7 cases with CR-PKIF. In contrast, post-2015 data showed a reversal, with 20 cases of open reduction and 32 cases utilizing CR-PKIF, as detailed in Table 7. Furthermore, the post-2015 assessment of elbow function outcomes, evaluated using the Mayo criteria, revealed a statistically significant improvement (*P* < 0.05) favoring CR-PKIF, suggesting enhanced recovery of elbow function with this minimally invasive technique. These findings collectively endorse the increasing adoption of closed reduction with percutaneous Kirschner wire internal fixation (CR-PKIF) as a preferred treatment modality for distal humeral intercondylar fractures in skeletally immature patients, highlighting its potential to become the future standard in this clinical context.

**Table 7:** Comparison of mayo elbow performance scores before and after 2015 in children and adolescents with AO/OTA type C2 distal humeral intercondylar fractures.

Year	Excellent	Good	Fair
Before 2015	27/46 (58.7%)	16/46 (34.8%)	3/46 (6.5%)
After 2015	41/52 (78.8%)	11/52 (21.2%)	0/52 (0%)

Chi-square test was performed (χ^2^ = 6.465, *P* = 0.039*), indicating a statistically significant difference in outcomes between the periods (*P* < 0.05).

* *P* < 0.05 denotes statistical significance.

This study acknowledges several limitations. Firstly, the small sample size in this study, particularly in the open reduction and internal fixation (ORIF) group, may compromise statistical power and restrict the ability to conduct subgroup analyses. Consequently, it fails to adequately characterize the epidemiological features of intercondylar fractures of the distal humerus in children and determine the optimal treatment strategy. Validation of these findings requires multi-center, large-sample studies. Secondly, at the time of the final follow-up, some patients had not achieved skeletal maturity (e.g., those ≤12 years of age). The risks of long-term growth disturbance and angular deformities (cubitus varus/valgus) necessitate prolonged follow-up for evaluation. Future studies should incorporate bone age monitoring and follow-up of more than 10 years to assess late-stage degenerative changes and functional outcomes. Thirdly, statistical analysis revealed partial or multiple data deficiencies in the reviewed literature, which may introduce bias into the study's conclusions. Augmenting existing data with high-volume case series will be crucial for generating higher-level evidence to inform clinical decision-making.

In summary, as a retrospective exploratory study, this research preliminarily demonstrates the feasibility of closed reduction and percutaneous pinning (CRPP) in selected cases, rather than recommending it as a universal treatment strategy. The management of intercondylar fractures of the distal humerus in children and adolescents remains controversial; however, in recent years, closed reduction and percutaneous fixation has achieved satisfactory reduction of fracture fragments and articular surfaces, along with the therapeutic goal of rapid functional recovery of the elbow joint. Combined with a systematic literature review, the excellent and good rate of elbow joint function has improved with the increasing application of CRPP. CRPP demonstrates non-inferiority to ORIF in achieving functional recovery, with advantages in operative efficiency for select cases.

## Data Availability

The raw data supporting the conclusions of this article will be made available by the authors, without undue reservation.
